# Farm management, not soil microbial diversity, controls nutrient loss from smallholder tropical agriculture

**DOI:** 10.3389/fmicb.2015.00090

**Published:** 2015-03-04

**Authors:** Stephen A. Wood, Maya Almaraz, Mark A. Bradford, Krista L. McGuire, Shahid Naeem, Christopher Neill, Cheryl A. Palm, Katherine L. Tully, Jizhong Zhou

**Affiliations:** ^1^Department of Ecology, Evolution and Environmental Biology, Columbia UniversityNew York, NY, USA; ^2^Agriculture and Food Security Center, The Earth Institute, Columbia UniversityNew York, NY, USA; ^3^Department of Ecology and Evolutionary Biology, Brown UniversityProvidence, RI, USA; ^4^School of Forestry and Environmental Studies, Yale UniversityNew Haven, CT, USA; ^5^Department of Biology, Barnard College of Columbia UniversityNew York, NY, USA; ^6^The Ecosystems Center, Marine Biological LaboratoryWoods Hole, MA, USA; ^7^Department of Plant Science and Landscape Architecture, University of MarylandCollege Park, MD, USA; ^8^Institute for Environmental Genomics and Department of Microbiology and Plant Biology, University of OklahomaNorman, OK, USA; ^9^Earth Science Division, Lawrence Berkeley National LaboratoryBerkeley, CA, USA; ^10^State Key Joint Laboratory of Environment Simulation and Pollution Control, School of Environment, Tsinghua UniversityBeijing, China

**Keywords:** carbon mineralization, denitrification, fertilization, GeoChip, microbial diversity, smallholder agriculture, tropics

## Abstract

Tropical smallholder agriculture is undergoing rapid transformation in nutrient cycling pathways as international development efforts strongly promote greater use of mineral fertilizers to increase crop yields. These changes in nutrient availability may alter the composition of microbial communities with consequences for rates of biogeochemical processes that control nutrient losses to the environment. Ecological theory suggests that altered microbial diversity will strongly influence processes performed by relatively few microbial taxa, such as denitrification and hence nitrogen losses as nitrous oxide, a powerful greenhouse gas. Whether this theory helps predict nutrient losses from agriculture depends on the relative effects of microbial community change and increased nutrient availability on ecosystem processes. We find that mineral and organic nutrient addition to smallholder farms in Kenya alters the taxonomic and functional diversity of soil microbes. However, we find that the direct effects of farm management on both denitrification and carbon mineralization are greater than indirect effects through changes in the taxonomic and functional diversity of microbial communities. Changes in functional diversity are strongly coupled to changes in specific functional genes involved in denitrification, suggesting that it is the expression, rather than abundance, of key functional genes that can serve as an indicator of ecosystem process rates. Our results thus suggest that widely used broad summary statistics of microbial diversity based on DNA may be inappropriate for linking microbial communities to ecosystem processes in certain applied settings. Our results also raise doubts about the relative control of microbial composition compared to direct effects of management on nutrient losses in applied settings such as tropical agriculture.

## INTRODUCTION

Agricultural management, such as mineral nutrient addition, can lead to marked changes in the taxonomic composition of soil microbial communities (Ramirez et al., 2010, 2012; Fierer et al., 2011; Wood et al., 2015). The pairing of mineral and organic nutrient addition to agriculture can significantly impact the ability of soil microbial communities to catabolize a range of carbon (C) substrates as well as affect the abundance of microbial functional genes involved in multiple aspects of C, nitrogen (N), and phosphorus (P) cycling (Wood et al., 2015). Some of the microbially driven processes associated with these changes in functional capacity, such as denitrification and decomposition, determine the retention and loss of nutrients in ecosystems and are thus important to managing agriculture for crop production while minimizing nutrient losses to the environment (Vitousek et al., 2009). There is thus keen interest in whether changes in microbial community composition can directly impact rates of ecosystem processes (e.g., Wessén et al., 2011; Wallenstein and Hall, 2012; Philippot et al., 2013; van der Heijden and Wagg, 2013; Krause et al., 2014).

Certain ecosystem processes are likely to be more sensitive to changes in microbial community composition than others. Narrow processes are most likely to be affected by changes in community composition because they require a specific physiological pathway and/or are carried out by a phylogenetically clustered group of organisms (Schimel and Schaeffer, 2012). Thus, processes can be either physiologically narrow, phylogenetically narrow, or both. In this manuscript we use the term “narrow” to refer to physiologically narrow processes that require specific physiological pathways, regardless of their distribution in the microbial phylogeny. For instance, we refer to denitrification as a narrow process because it requires particular genes that code for enzymes capable of reducing various forms of nitrogen. Because a relatively small proportion of microorganisms carry these genes, changes in community composition that lead to a shift in the relative abundance of denitrifiers—or changes in the abundances of the relevant functional genes—should have significant impacts on rates of denitrification (Pett-Ridge and Firestone, 2005; Philippot et al., 2013; Powell et al., 2015). Mineralization of soil C to CO_2_, by contrast, is a broad process because the ability to mineralize and respire C substrates is relatively simple and shared by many microbial taxa (Schimel and Schaeffer, 2012). We thus expect that carbon mineralization would not respond strongly to changes in the composition of microbial communities.

Whether this framework of broad and narrow processes helps predict nutrient losses from agriculture depends on the relative importance of the multiple potential drivers of ecosystem process rates, including microbial community composition, nutrient availability, and soil and environmental properties. Though several studies have found support for microbial influence on narrow processes, such as denitrification, such studies often focus on identifying whether microbial community composition is related to ecosystem processes, but stop short of quantifying the relative contribution of the multiple controls on ecosystem processes (e.g., Philippot et al., 2013). Understanding the importance of biodiversity requires assessing the influence of composition relative to other biotic and abiotic controls (Laliberté and Tylianakis, 2012; Bradford et al., 2014).

Following theory (Schimel, 1995; Schimel and Schaeffer, 2012), we hypothesize that changes in microbial diversity will have a stronger effect on denitrification than will the direct effect of nutrient addition—measured as both N addition and the inclusion of seasonal legume rotations (henceforth *agroforestry*) to increase soil C—if changes in diversity correspond with changes in the relative abundance of denitrifying taxa and the abundances of functional genes involved in denitrification. Because C mineralization is a broad process, we expect that nutrient addition will have a stronger effect on process rates than changes in the microbial community.

## MATERIALS AND METHODS

### SITE SELECTION

**FIGURE 1:**
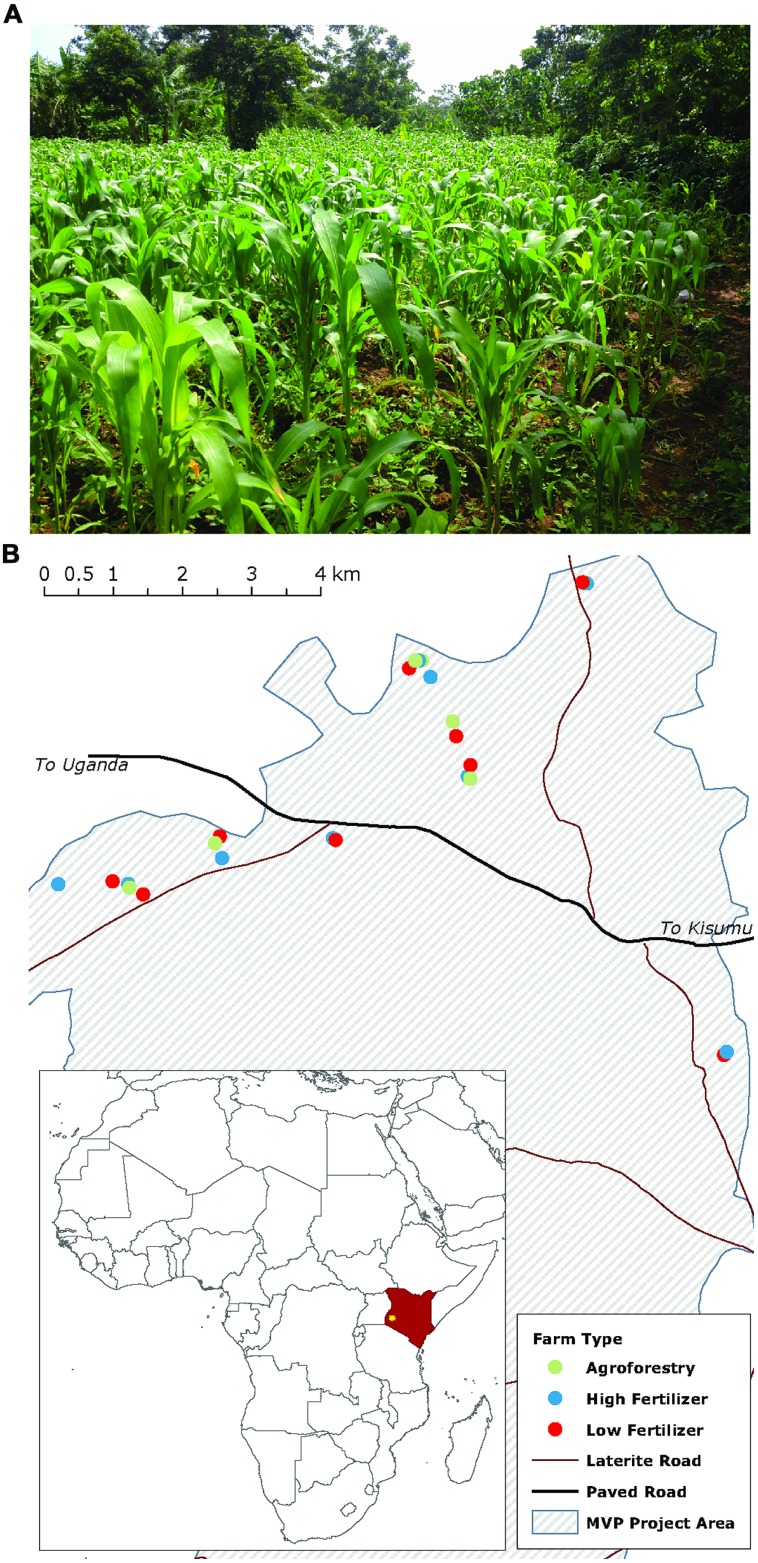
**Maize production in western Kenya mainly occurs on smallholder farms of around 1 hectare **(A)**.** Map **(B)** shows the study farms and their distribution across the Millennium Villages Project site area in western Kenya. Farm types are coded by color.

We examine our hypotheses on 24 smallholder farms in western Kenya participating in the Millennium Villages Project (MVP) site in Sauri, Kenya (**Figure 1**; Wood et al., 2015). The center of the study area is located at 0∘06′04.88 N, 34∘30′40.12 E at an elevation of 1450 m. The mean annual temperature and precipitation for the study region are 24∘C and 1800 mm, respectively. Annual precipitation is distributed bi-modally with 1120 mm in a long rainy season from March to June and 710 mm in a short rainy season from September to December. The soils are classified as Oxisols and are well drained sandy clay loams (on average 53.75% sand, 12.59% silt, 33.54% clay) with a mean pH of 5.45 and C:N of 11.52 (0–20 cm). The study zone was originally part of the moist broadleaf forest area in eastern and central Africa, but is now a mixed-maize agricultural system, with most farmers cultivating maize in both the long and short rainy seasons. Some farmers, however, replace the short rain maize crop with a seasonal legume rotation that fixes nitrogen and builds soil organic matter.

The MVP was designed to meet the Millennium Development Goals at the village scale in Sub-Saharan Africa and includes an agricultural component that focuses on increasing crop yields through mineral and organic nutrient addition to redress negative soil nutrient balances (Sanchez et al., 2007). This is primarily achieved by subsidizing mineral fertilizers (primarily diammonium phosphate and urea). Farmers are also trained in seasonal legume rotations to fix nitrogen and build soil organic matter. In Sauri, rotational legume trainings have been promoted since the early 1990s (Kiptot et al., 2007) and fertilizer subsidy programs were active from 2005 to 2008.

We selected farms to participate in the study based on 2 years of household surveys. We determined nutrient inputs and outputs for each of these farms through a combination of interviews, on-farm crop harvests, and biomass estimations. Farms were classified into three categories: *low fertilizer*, *high fertilizer*, and *high fertilizer + agroforestry* (specifically, seasonal legume rotations). Low fertilizer farms have applied less than 10 kg mineral N ha^-1^ y^-1^ since 2005; high fertilizer farms have applied at least 60 kg N ha^-1^ y^-1^ over the same time period. High fertilizer + agroforestry farms (henceforth *agroforestry*) apply amounts of mineral N comparable to *high fertilizer* farms, but also use agroforestry techniques to build soil organic matter. These agroforestry techniques replace short-rain maize crops with fast-growing leguminous tree, shrub, or herbaceous species that are planted from seed and cut each year for organic inputs to crop fields. These techniques are referred to generally as agroforestry, though agroforestry is a general term that captures different practices not studied here (e.g., wind breaks, live fencing, etc.). Our results, therefore, apply to agroforestry strategies that seasonally incorporate legume rotations.

We estimated the amount of N added to farms with farmer-reported data on the quantity of N added through mineral and organic sources (diammonium phosphate, urea, biological N_2_-fixation, and manure). For agroforestry farms, we also estimated the amount of N added through N_2_-fixation based on both literature-reported values and field-reported biomass estimates. To estimate the amount of N added through N_2_-fixation we collected data on legume species planted, original planting density, thinning practices, wood harvesting, and legume management. We used plant density to estimate the amount of aboveground biomass N for each species present and then used literature data on the percent of total N derived from biological N_2_-fixation for each species to calculate the amount of N derived from fixation (Gathumbi et al., 2002a,b; Ojiem et al., 2007). Because farmers tend to remove woody stems but incorporate fresh leaves, we removed the amount of N stored in woody biomass from this value to estimate the net N contribution from the legume species to the farm fields. We conservatively estimate that N_2_-fixation contributed between 30 and 50 kg N ha^-1^ year^-1^ during the short rain fallow, up to 30 kg of which may be due to the presence of *Mucuna pruriens*, an annual climbing legume (Ojiem et al., 2007). Planting densities, however, can vary widely from year-to-year with low-density years being as low as an order of magnitude less than those assumed in this estimate. Thus, depending on the year, actual fixation rates may be as low as 5–30 kg N ha^-1^ short rainy season^-1^. We use the term ‘nutrient addition’ to refer to both N addition on low- and high-fertilizer and agroforestry farms as well as C addition through agroforestry. The final farms included in the study are distributed across the Sauri village cluster, but are clustered by treatment (**Figure 1**) on similar underlying soils.

### SAMPLE COLLECTION AND MEASUREMENT

Soil sampling was conducted in June 2012, in the middle of the long rains, 2 weeks after fertilizer application. On the farm fields, we took 15 2-cm diameter soil cores from the top 20 cm of bulk soil. Cores were taken at regular intervals throughout the entire farm field and homogenized and aggregated to a composite sample. At each core location we recorded temperature and volumetric soil moisture content using a soil thermometer and a HydroSense moisture probe (Campbell Scientific, Logan, UT, USA). We sieved soils to 2 mm and stored soil for DNA extraction at -20∘ C. Soils for DNA extraction were transported to the U.S. within 1 week of sampling. Subsamples of sieved field soil were stored at 4∘ C, transported to the U.S. within 1 week of sampling, and used to determine pH, gravimetric soil moisture, and water holding capacity. Gravimetric soil moisture and water holding capacity (after wetting soils to field capacity) were determined by drying soil at 105∘C for 24 h. Soil pH was determined using a benchtop meter of a 1:1 slurry of soil:H2O by volume.

A subsample of sieved soil was air-dried and used to determine total C and total N by combustion with an Elementar Vario Macro CNS analyzer. Total extractable P was assessed by combining a 5-g soil sample with 20 mL of Mehlich I extraction solution and shaking for 5 min followed by inductively coupled plasma spectrometry (Varian Vista MPX Radial ICP-OES). Soil nutrient assays were performed at the Auburn University Soil Testing Laboratory (AL, USA). Sieved, air-dried soil was also used to determine soil texture using the hydrometer method that uses sodium hexametaphosphate to complex the anions that bind to clay and silt particles into aggregates and suspend organic matter in solution. The density of the soil suspension is determined using a hydrometer after the sand particles settle and then after the silt particles settle (Bouyoucos method).

Denitrification and C mineralization assays were performed in Kenya on fresh soils at the MVP regional office in Kisumu, Kenya. Denitrification potential was estimated based on N_2_O emissions during denitrifying enzyme activity (DEA) assays (Smith and Tiedje, 1979). In a 125-mL flask, we combined 20 g of soil with 20 mL of a 1-mM sucrose and 1-mM KNO_3_^-^ solution. We fit each flask with a #5 stopper, which was inserted with a 22G needle capped with a stopcock. We then brought the headspace of the flask to 10% acetylene (C_2_H_2_) concentration by volume (to inhibit the reduction of N_2_O to N_2_ via denitrification). At the beginning of the incubation we closed the stopcocks and placed the flasks onto a shaker table at 125 rpm; flasks were only removed from the table for sampling. We sampled the headspace five times: at 30, 150, 210, and 270 min, by removing 30 mL of gas from the headspace and then replacing the volume of headspace that was removed with 30 mL of 10% C_2_H_2_ room air (fluxes were corrected for N_2_O molecules removed at each sampling period). DEA headspace samples were stored in pre-evacuated vials.

Water-amended soil incubations were used to measure CO_2_ eﬄux and, thus, actual C mineralization. These incubations were performed identically to the DEA incubations with three exceptions: (1) 20 mL of deionized water was added to soil in place of the sucrose and KNO_3_^-^ solution; (2) no C_2_H_2_ was added to the headspace; and (3) headspace samples were taken at only two time points (240 and 360 min). We also sampled room air at the beginning and end of each incubation and included travel standards to accompany samples, in order to correct for any sample loss during transport and storage. DEA and CO_2_ headspace samples were transported to the U.S., where we determined N_2_O and CO_2_ concentrations by gas chromatography using a Shimadzu GC-14 GC with electron capture (for N_2_O) and thermal conductivity (for CO_2_) detectors at the Cary Institute (Millbrook, NY).

To measure taxonomic diversity, we performed 16S rRNA amplicon sequencing of bacteria and archaea following standard protocols of the Earth Microbiome Project using an Illumina MiSeq instrument ( ^1^Gilbert et al., 2010; Caporaso et al., 2012). Briefly, we extracted DNA using a MoBio PowerSoil 96-well extraction kit and we amplified the 16S rRNA V4 gene from bacterial and archaeal genomes using the primers 515F (forward) and 806R (reverse; Caporaso et al., 2012). The 16S rRNA gene is a well-conserved gene in bacteria and thus captures evolutionary relationships among bacterial taxa. Quality filtering was performed by comparing input sequences with Phred scores (Q ≥ 20). Sequences shorter than 75% of the Phred score were discarded as well as sequences with ambiguous base call characters. All quality filtering and demultiplexing were performed using the split_libraries_fastq.py algorithm in QIIME and its associated default parameters (^1^Caporaso et al., 2010). Sequence reads were were binned into operational taxonomic units (OTUs) at a 97% similarity threshold. OTUs were then compared to GenBank to identify bacterial lineages. A total of 3,462,835 bacterial sequences were generated across all samples, representing 29,195 OTUs. Sequence lengths averaged 150.63 ± 2.93 per sample. Rarefaction was used to compare samples at depth of 40 sequences per sample. We calculated taxonomic diversity as Shannon diversity (H’) of all OTUs. We calculated other diversity metrics, such as Faith’s PD, and found similar patterns. All data checks and processing were done using QIIME (Caporaso et al., 2010).

To estimate microbial functional diversity, we measured the abundance of key functional genes using GeoChip 4.0 to analyze DNA samples that were extracted following the protocol for taxonomic assessment. GeoChip is a functional gene array of bacteria, archaea, and fungi covering 401 gene categories involved in major biogeochemical and ecological processes, as previously described (He et al., 2007; Yang et al., 2013; Tu et al., 2014). GeoChip examines the abundance of thousands of functional gene variants simultaneously through a fluorescent procedure. DNA samples were labeled with a fluorescent dye and purified using a QIA quick purification kit (Qiagen, Valencia, CA, USA) following He et al. (2007) and Tu et al. (2014). DNA was then dried in a SpeedVac (ThermoSavant, Milford, MA, USA) and labeled DNA was resuspended in a hybridization solution before hybridization of DNA was carried out on a MAUI hybridization station (BioMicro, Salt Lake City, UT, USA). GeoChip microarrays were scanned by a NimbleGen MS200 scanner (Roche, Madison, WI, USA). Poor quality spots were removed when flagged as one or three by ImaGene (Arrayit, Sunnyvale, CA, USA) or with a signal-to-noise ratio of less than 2.0. Signal-to-noise ratio indicates the amount of luminescence from the sample compared to background noise. Average signal-to-noise ratios are often greater than 50 (He et al., 2007), so 2.0 represents high noise to signal. Processed data were subjected to the following steps: (i) normalize the signal intensity by dividing the signal intensity by the total intensity of the microarray followed by multiplying by a constant; (ii) transform by the natural logarithm; (iii) remove genes detected in only one out of three samples from the same treatment. Signal intensities were quantified and processed using a previously described data analysis procedure (He et al., 2007; Yang et al., 2013). We calculated functional diversity as Shannon diversity (H’) of the signal intensity for all of the genes reported from the array. We also analyzed the response of individual denitrification genes to changes in functional diversity. These include genes involved in nitrite reduction (*nirK*, *nirS*), nitrate reduction (*narG*), and nitric oxide reduction (*norB*). GeoChip also includes *nosZ*, which is involved in nitrous oxide reduction, but we do not analyze this gene because it is involved in a later stage of denitrification than represented by the denitrification potential assay.

### DATA ANALYSIS

We used structural equation models to simultaneously estimate each of the pathways among nutrient addition, soil and environmental properties (pH, texture, and moisture), microbial communities, and ecosystem processes while accounting for correlations between multiple response variables (Grace, 2006). Structural equation modeling is increasing used in ecology and environmental sciences to assess the relative impacts of multiple variables on each other and a set of response variables (Grace, 2006). This technique has been applied to a wide range of issues in ecology and environmental sciences (e.g., Byrnes et al., 2011; Flynn et al., 2011; Laliberté and Tylianakis, 2012). Relevant to our study, it was used by Colman and Schimel (2013) to determine the drivers of microbial respiration and N mineralization at continental scales.

To test our hypotheses about the relative importance of nutrient addition and microbial composition, we first fitted models including both nutrient addition and microbial diversity variables. Soil pH was the only significant environmental control and was thus the only environmental variable retained in the final models. We then fitted models to optimize goodness-of-fit and do not include variables that do not contribute strongly to model goodness-of-fit. Different models were fitted for each of the two response variables (denitrification potential and C mineralization). For each response variable, constrained (microbial + nutrient addition) and unconstrained models were compared based on change in AIC values. The final, unconstrained model retained nutrient addition and pH, but did not include microbial diversity.

We report standardized path estimates that allow for comparison of the relative magnitude of variables within the same model (Grace and Bollen, 2005). For model goodness-of-fit, we report *X*^2^ and root mean square error of approximation (RMSEA). These measures assess the similarity between the covariance matrix of the observed data and the covariance matrix implied by the specified model. A *X*^2^ *P*-value greater than 0.05 implies significant overlap between the observed and implied data, and thus adequate model fit. We report Sartorra-Bentler *X*^2^ correction factors to improve estimates based on violations of multivariate normality. Because the *X*^2^ test is based on large sample theory, we also report RMSEA, which is a goodness-of-fit measure weighted by sample size. We use an RMSEA value below 0.1 to represent good model fit because for sample sizes less than 50, the conventional RMSEA cut-off value of 0.05 is overly conservative (Chen et al., 2008). Individual paths were estimated using maximum likelihood and we considered paths to be significant at *P* < 0.05 and marginally significant at *P* < 0.10 (Hurlbert and Lombardi, 2009). Insignificant paths were excluded from models unless they significantly improved overall model fit, based on *X*^2^ and RMSEA values as well as assessment of modification indices (Grace, 2006). All models were fitted using the *lavaan* package in R (Rosseel, 2012).

## RESULTS

We hypothesized that changes in microbial diversity would have a stronger effect on denitrification than would the direct effect of nutrient addition if changes in diversity correspond with changes in the relative abundance of denitrifying taxa and/or the abundance of associated genes involved in denitrification. We also hypothesized that nutrient addition would be a stronger predictor of C mineralization, a broad process, than microbial diversity.

**FIGURE 2:**
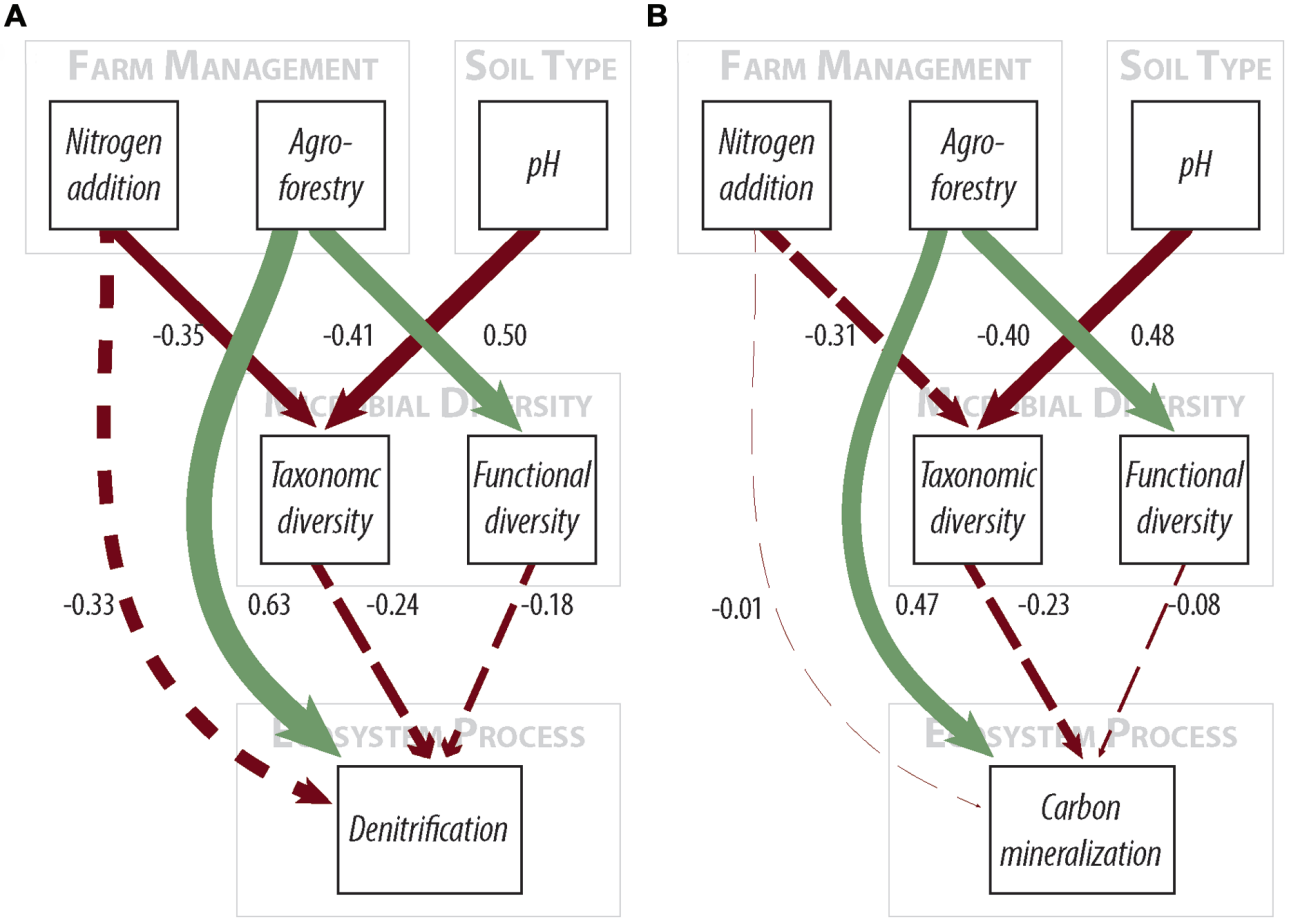
**Path diagrams for structural equation models of the relationship between farm management, microbial diversity, and **(A)** denitrification enzyme activity or **(B)** carbon mineralization.** Models **(A,B)** show the relative effect of management and microbial diversity. Solid paths are statistically significant at *p* < 0.10. Dashed paths are insignificant, but were included for hypothesis testing or overall model fit. Line color represents effect direction (light green = positive, deep red = negative). Path widths are proportional to standardized regression coefficients, which are shown next to each path. Results and model statistics are in **Table 2**.

**FIGURE 3:**
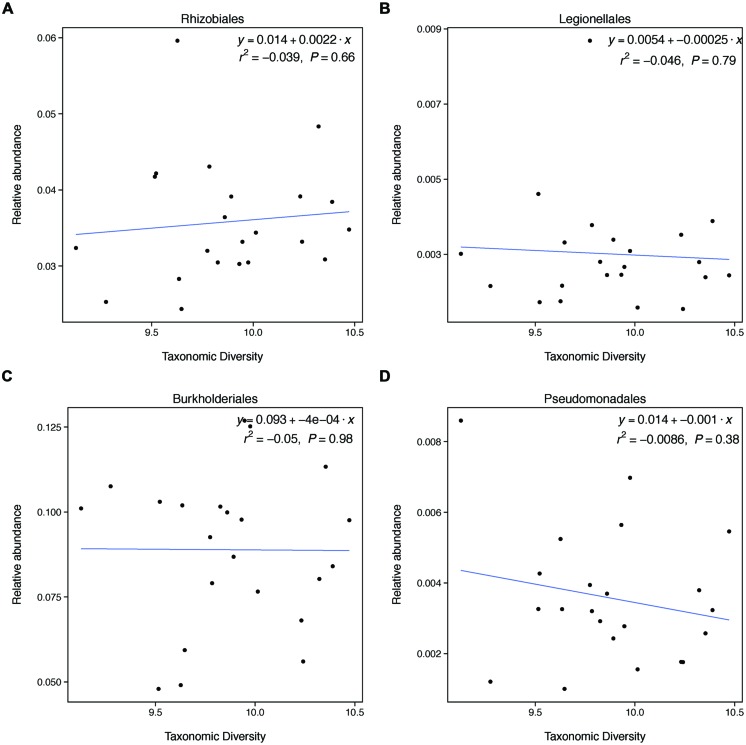
**Taxonomic diversity is not related to changes in the relative abundances of select denitrifying taxa.** These groups do not represent all categories of denitrifying taxa and not all taxa within these categories are able to carry out denitrification. These groups were selected because they broadly represent evolutionary lineages that are capable of denitrification and had relatively high relative abundances in our samples.

**FIGURE 4:**
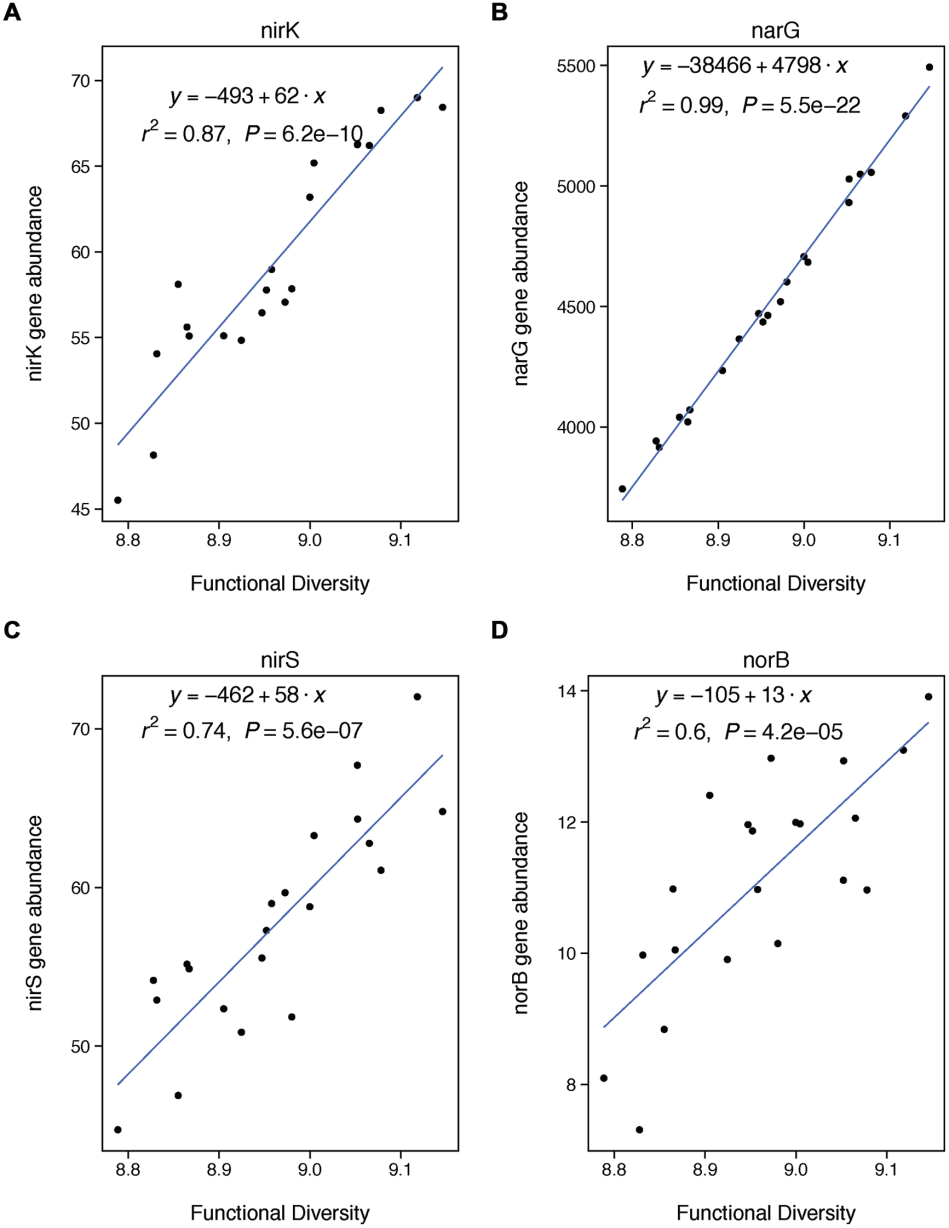
**Functional diversity is positively correlated with changes in the abundances of specific genes involved in denitrification.** These genes are involved in nitrite reduction (**A**: nirK, **C**: nirS), nitrate reduction (**B**: narG), and nitric oxide redunction (**D**: norB). We did not analyze nosZ because it is involved in a later stage of denitrification than included in our potential assay (nitrous oxide reduction).

We find that farm management—through N addition and agroforestry—impacts the taxonomic and functional diversity of soil microbial communities. Specifically, taxonomic diversity decreases by 2.40% from low-to-high N addition (**Table 1**), though this effect is weaker than the effect of pH, which is also associated with lower taxonomic diversity (**Figures 2A,B**). We did not find that these changes in taxonomic diversity were coupled with changes in the relative abundance of select groups of denitrifying taxa (**Figure 3**). Agroforestry was the strongest driver of functional diversity, which increased 1% between high fertilizer and agroforestry farms and 2% between low fertilizer and agroforestry farms (**Table 1**; **Figures 2A,B**). We did find that greater functional diversity is significantly related to greater abundances of several genes involved in denitrification: *nirK*, *nirS*, *norB*, and *narG* (**Figure 4**).

**Table 1:** Means and SD for variables included in structural equation models among the three categories of nutrient addition: low fertilizer, high fertilizer, and agroforestry.

Farm type	Denitrification *(ng N g dry soil^-1^ h^-1^)*	C mineralization *(ug C g dry soil^-1^ h^-1^)*	Taxonomic diversity	Functional diversity	Sand	Silt*%*	Clay	pH*log[H^+^]*	C*%*	N*%*	P*ppm*
			H’								
Low fertilizer	0.61 [0.49]	1.04 [0.24]	10.02 [0.31]	8.88 [0.07]	53.76 [5.64]	14.40 [7.61]	31.74 [6.34]	5.41 [0.35]	1.83 [0.20]	0.20 [0.03]	16.63 [9.15]
High fertilizer	0.48 [0.09]	0.99 [0.41]	9.78 [0.45]	8.99 [0.08]	56.00 [3.13]	9.71 [5.91]	34.15 [6.57]	5.06 [0.37]	1.95 [0.16]	0.22 [0.03]	19.13 [10.30]
Agroforestry	1.00 [0.58]	1.27 [0.13]	9.79 [0.30]	9.05 [0.09]	58.58 [2.06]	10.46 [4.67]	30.86 [4.96]	5.47 [0.72]	1.72 [0.27]	0.18 [0.02]	7.00 [2.55]

*All soil properties are to a depth of 20 cm. Because of unbalanced design statistical comparisons between groups are not valid; instead the effect of Farm type is represented by the path coefficients of Agroforestry and N Addition in the structural equation models. Further detail on changes in soil properties is presented in Wood et al. (2015).*

**Table 2:** Model results and goodness of fit statistics for structural equation models.

Denitrification					C Mineralization				
		*Standardized estimate*	*P*				*Standardized estimate*	*P*
Denitrification∼					C mineralization∼			
	Agroforestry	0.63	0.00			Agroforestry	0.47	0.00
	Functional diversity	-0.18	0.31			Functional diversity	-0.08	0.72
	N addition	-0.33	0.10			N addition	-0.01	0.95
	Taxonomic diversity	-0.24	0.18			Taxonomic diversity	-0.23	0.35
Taxonomic diversity∼					Taxonomic diversity∼			
	N Addition	-0.35	0.06			N Addition	-0.31	0.18
	pH	-0.41	0.00			pH	-0.40	0.01
Functional diversity∼					Functional diversity∼			
	Agroforestry	0.50	0.01			Agroforestry	0.48	0.03
**Structural equation model metrics**						**Structural equation model metrics**			
	*n*		21			*n*		21
	*df*		5			*df*		5
	χ^2^		2.14			χ^2^		2.62
	P_χ2_		0.83			P_χ2_		0.76
	RMSEA		0.00			RMSEA		0.00
	P_ RMSEA_		0.85			P_RMSEA_		0.75

*We report robust *X*^2^ statistics for model fit. *P* > 0.05 indicates that estimated models have covariance matrices among variables that are not strongly different from observed values and that the model, therefore, adequately represents the data. Root mean square error of approximation (RMSEA) is a sample-size weighted measure of model fit. Values below 0.1 indicate good model fit.*

We did not, however, find that changes in taxonomic and functional diversity were related to rates of either denitrification or C mineralization. Instead, ecosystem process rates were most strongly linked to the direct effect of farm management. Denitrification decreased by 21.31% from low-to-high N and increased by 63.93% from low N to agroforestry (**Table 1**). The path estimate for agroforestry on denitrification (0.63) is three times greater than the coefficient for either taxonomic diversity (-0.24) or functional diversity (-0.18). The agroforestry coefficient is also twice the magnitude of the coefficient for N addition (-0.33). We find support for our hypothesis that C mineralization will be more influenced by nutrient addition than microbial community composition. C mineralization rates were 4.81% lower on high-vs.-low N farms and 22.12% greater under agroforestry (**Table 1**). The path coefficient for the effect of agroforestry on C mineralization (0.47) is more than twice as great as the coefficient for taxonomic diversity (-0.23) and N addition (0.16) and around five times the effect of functional diversity (-0.08).

## DISCUSSION

Our results reveal that shifts in microbial taxonomic and functional diversity due to farm management are not significantly related to either denitrification or C mineralization on smallholder farms in western Kenya. This finding supports our hypothesis that C mineralization would not be sensitive to changes in microbial communities because it is a broad process that can be carried out by many microbial taxa. However, we did not find support for our hypothesis that denitrification would be sensitive to community change because it is a narrow process carried out by relatively few taxa.

This unexpected result may be explained by the fact that changes in taxonomic diversity were not coupled with decreases in the relative abundance of denitrifying taxa. Our hypothesis was built on the expectation that diversity would relate to denitrification rates if changes in diversity were paired with changes in the relative abundance of taxa able to carry out denitrification. Because denitrifying taxa are found widely through the microbial phylogeny, it is difficult to identify groups of taxa that are all denitrifiers. However, we found that groups that broadly contain denitrifiers do not change in relative abundance with changes in diversity. This finding may explain why taxonomic diversity was not a significant predictor of denitrification.

We also expected that functional diversity would be a significant control on denitrification if changes in functional diversity were coupled with changes in the abundances of key denitrifying genes. We did find a strong coupling between our functional diversity metric (Shannon diversity of all functional genes from GeoChip) and the abundances of four particular genes key to denitrification: *nirK*, *nirS*, *narG*, and *norB*. Thus, our finding that functional diversity was not significantly related to rates of denitrification was unexpected. However, the finding fits with recent meta-analysis showing that microbial functional gene abundances are rarely strongly correlated with corresponding process rates (Rocca et al., 2014). Our lack of observed relationship between gene abundances and process rates may be explained by the fact that our measure of functional diversity is based on the presence of functional genes using DNA. Because DNA only indicates the presence of a gene, rather than whether that gene is expressed, our measure of functional diversity only represents a coarse picture of microbial functional capacity. Our results thus suggest that rates of denitrification are more strongly controlled by the expression of functional genes, rather than their overall abundance. This finding suggests that coarse measures of microbial communities based on DNA—whether taxonomic or functional—may be insufficient to understanding the changes in the functional contributions of these communities under certain types of land management (Rocca et al., 2014).

Though understanding when microbial communities should impact ecosystem process rates is well established, we show that actual changes in microbial communities observed in a tropical agroecosystem are not a strong predictor of denitrification rates because changes in microbial communities are relatively minor in magnitude. Our findings, however, do not invalidate the hypothesis that narrow processes are sensitive to community composition and broad processes are not, which has been supported in previous work (e.g., Salles et al., 2012; Schimel and Schaeffer, 2012; Philippot et al., 2013; Powell et al., 2015). Instead, our findings raise doubts about the relative importance of microbial community composition compared to direct effects of nutrient addition on nutrient losses in agricultural settings. This is because the magnitude of change in microbial diversity induced by land management was not large enough to significantly impact ecosystem process rates. As a result, the direct effect of farm management (rather than the indirect effect through changes in microbial communities) was the dominant control of both denitrification and C mineralization. Whether changes in microbial community composition translate into changes in rates of ecosystem processes controlled by soil microbes is of great interest in soil ecology (e.g., Torsvik and Øvreås, 2002; Philippot and Hallin, 2005; van der Heijden et al., 2008), but remains an ongoing debate (Schimel and Schaeffer, 2012). Our study is unique, however, in that few studies have connected changes in microbial communities to ecosystem process rates in a framework that assesses the relative importance of the multiple drivers of these ecosystem processes.

Although we focus on smallholder farms in western Kenya, there is a widespread effort to increase crop yields across sub-Saharan Africa and in tropical smallholder agriculture more generally (Wiggins et al., 2010). Because 75% of the world’s 1.2 billion poorest people are engaged in smallholder, making up 500 million farms of less than 2 ha (Wiggins et al., 2010), our findings may help inform understanding of drivers of nutrient loss in tropical smallholder agriculture due to increased attention from international development organizations.

It is becoming widely recognized that agricultural sustainability requires agricultural practices that maximize multiple ecosystem services while minimizing nutrient losses to the environment (Foley et al., 2011; Bommarco et al., 2013). This is particularly important in tropical ecosystems that are undergoing large-scale modifications of nutrient cycling pathways due to intense efforts by the international development community to increase fertilizer use by tropical smallholder farmers. Further work should focus on understanding how management-induced shifts in microbial communities impact not just potential nutrient loss, but the multiple ecosystem services provided by soil and how such understanding can be integrated into sustainable agricultural strategies.

## AUTHOR CONTRIBUTIONS

SAW and MA conceived research and performed lab and field work; SAW, MA, MAB, KLM, SN, CN, CAP, and KLT designed the study; JZ performed GeoChip analyses; SAW analyzed data and wrote the first draft of the manuscript; all authors contributed to interpretation of results and commented on the manuscript. The authors declare no conflicts of interest.

## Conflict of Interest Statement

The authors declare that the research was conducted in the absence of any commercial or financial relationships that could be construed as a potential conflict of interest.

## Footnotes

1
http://www.earthmicrobiome.org/emp-standard-protocols/
