# Supplementary material for: Farm management, not soil microbial diversity, controls nutrient loss from smallholder tropical agriculture
Source: Front Microbiol. 2015 Mar 4;6:90. doi: 10.3389/fmicb.2015.00090 (PMC4396515; doi:10.3389/fmicb.2015.00090)
Supplement: Supplementary file 1 [file Table_1.DOCX]

**Supplementary Material**

**Tables**

**Table S1**. Farm selection information for high fertilizer, low fertilizer, and agroforestry farms. Data are based on two years of survey information regarding farm practices for the past ten years.

|  | **High Fertilizer** | **Low Fertilizer** | **Agroforestry** |
| --- | --- | --- | --- |
| Number of farms | 8 | 8 | 5 |
| Nutrient input type | Diammonium phosphate, urea, manure | Diammonium phosphate, urea, manure | Diammonium phosphate, urea, manure |
| Amount of N added | ~ 60 kg N ha^-1^ yr^-1^ | < 10 kg N ha^-1^ yr^-1^ | 65 - 110 kg N ha^-1^ yr^-1^ |
| Species present |  |  | *Calliandra calothyrsus Crotalaria grahamiana Crotalaria paulina Crotalaria ochroleuca Mucuna pruriens Tephrosia candida* |
| Plant density |  |  | 2000 plants ha^-1^ |
